# Practical Notes on Diagnosis and Treatment

**Published:** 1910-02-12

**Authors:** 


### Practical Notes on Diagnosis and Treatment.

**Dyspepsia.**

No case of purely functional dyspepsia can resist a pedestrian tour over the Alps.—*Sir James Johnston.*

**Aerated Milk.**

Where a change from ordinary milk is required for patients with feeble digestive power, milk charged with carbonic-acid gas will be found both palatable and refreshing.

**Renal Colic.**

Belladonna in large doses—30 to 40 minims every two or three hours—until slight delirium sets in, will often produce cessation of the pain and be followed by passage of the calculus.—*Dr. W. Murray.*

**Tinctura Laxativa.**

This is an agreeable and physiologically compounded laxative mixture: Two drachms each of liquid extract of cascara, aromatic spirit of ammonia, and spirit of chloroform, and one drachm each of the tinctures of belladonna and nux vomica. For chronic constipation a teaspoonful dose thrice daily, gradually diminished.

**Anæmia and the Nasal Mucosa.**

The mildest of the conditions in which there is dryness of the nasal mucous membrane is simple anæmia, where there is collapse of the erectile tissue, insufficient supply of blood to the mucous glands of the inferior turbinated bone, and consequent diminution of the secretion. This condition is not a disease, but simply a symptom of general anæmia.—*Dr. G. Macdonald.*

**Drip Enemata.**

By this method larger quantities of nourishment than are contained in the ordinary nutrient enemata may be administered with very little disturbance to the patient. The food is contained in a reservoir surrounded by hot water, and allowed to trickle through a soft tube, on which is a regulating clamp. In this way a pint or more may be given in an hour. The ingredients are as follows: Yolks of two eggs, 1 oz. of dextrose (pure), 30 grains of salt, and pancreatised milk to 15 ounces.

**Purgative Enema.**

An efficient purgative enema for obstinate cases is composed of one ounce each of mag. sulph., sodæ sulph., and glycerine, with 15 oz. of water.

**Bi-lateral Sciatica.**

True sciatica is rarely bi-lateral. Such cases always call for examination of the urine for diabetes, and the nervous system for evidence of tabes dorsalis or other spinal disease. Other causes, such as a pelvic tumour, must also be excluded.

**Gall-stones.**

Patients who suffer from symptoms of gall-stones very often do not drink enough. Advise them to drink a glass of hot water slowly one hour before every meal and also while dressing in the morning and while retiring at night. In this way the stomach is washed out preparatory for the next meal.—*Sir T. Lauder Brunton.*

**Sodæ Salicylas.**

This drug will be found useful in 5-grain doses in those numerous cases where "Mist. Gent. Alk." is usually given. The continued ingestion of bicarbonate of soda over long periods is likely to prove harmful, and so "repeats" should not be given indefinitely. Sodæ salicylas has, probably, some antiseptic action and is, moreover, a powerful direct chologogue. It may with advantage be combined with a few drops of tincture of nux vomica.

**Vomiting in Phthisis.**

Omitting cases of catarrhal gastritis, the most obstinate cases are those in which there is excessive irritability of the stomach. In these cases relief has frequently followed the use of liquor potassæ (1 to 5 minims) with calumba and a few minims of laudanum. This counteracts the excessive acidity of the stomach, which is in itself sufficient to keep up a certain amount of irritability of the gastric mucosa. A combination of creosote and opium is frequently successful. In intractable cases it is recommended to inject hypodermically in the epigastric region, one-sixth to one-fourth of a grain of morphia, the patient being kept in bed and in the recumbent position.—*Dr. S. H. Habershon.*

